# Infrared Multiple Photon Dissociation Spectroscopy of the H–H Stretching Mode and Low-Lying Electronic Transitions in Fe^+^(H_2_)_1,2_ and Fe^+^(D_2_)_1,2_

**DOI:** 10.1021/acs.jpca.5c00196

**Published:** 2025-04-02

**Authors:** Shan Jin, Marcos Juanes, Christian van der Linde, Milan Ončák, Martin K. Beyer

**Affiliations:** † Institut für Ionenphysik und Angewandte Physik, Universität Innsbruck, Technikerstraße 25, Innsbruck 6020, Austria; ‡ Departamento Química Física y Química Inorgánica, University of Valladolid, Paseo de Belén 7, Valladolid 47011, Spain

## Abstract

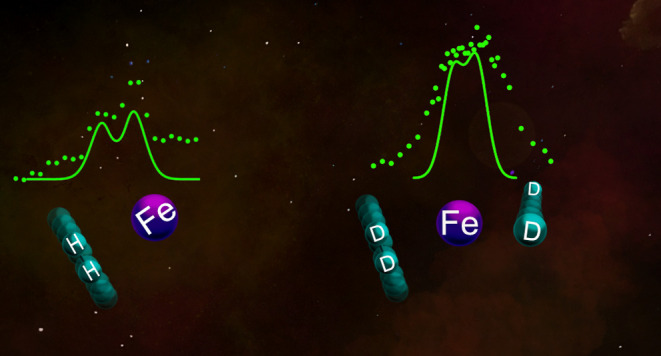

Although iron is the most abundant transition metal in the interstellar medium, its interaction with hydrogen—by far the most abundant element—in small gas-phase molecules or complexes is poorly understood. Herein, we study the infrared spectroscopy of cationic iron complexes with one and two dihydrogen ligands, Fe^+^(H_2_)_1,2_, as well as their deuterated counterparts, Fe^+^(D_2_)_1,2_, using infrared multiple photon dissociation (IRMPD) spectroscopy. Quantum chemical calculations, including multireference configuration interaction (MRCI) with spin–orbit coupling, are used to simulate the electronic and vibrational contributions to the spectra. Broad electronic transitions are observed in the studied energy range of 2230–4000 cm^–1^, which arise from d–d transitions at the metal center between states of quartet spin multiplicity. In the complex, the H–H stretching mode of the H_2_ ligand becomes infrared active, and features arising from this mode are assigned with the help of quantum chemical calculations in the spectra of Fe^+^(H_2_) and Fe^+^(D_2_)_2_. In Fe^+^(H_2_), we assign a band with local maxima centered at ∼3138 cm^–1^ and ∼3219 cm^–1^ to the P and R branches of the H–H stretching mode, while the D–D stretch of Fe^+^(D_2_)_2_ has a band centered at 2448 cm^–1^, with P and R branches not resolved. With a D/H wavenumber ratio of 0.726, the D–D stretch of Fe^+^(D_2_) and the H–H stretch of Fe^+^(H_2_)_2_ are expected at 2309 cm^–1^ and 3372 cm^–1^, respectively. The rovibrational bands in Fe^+^(H_2_) and Fe^+^(D_2_)_2_ exhibit pronounced broadening that cannot be explained by temperature. We assign the broadening to the strong dependence of the H–H and D–D stretching frequencies on the torsional motion of the complex, as shown by the calculations. The extreme redshift of the H–H and D–D stretching frequencies is caused by back-donation from iron d_*xz*_, d_*yz*_ atomic orbitals into the σ* orbital of the H_2_ molecule, which weakens the H–H bond.

## Introduction

As the most abundant molecule in dense interstellar clouds, H_2_ plays a critical role in astrochemical reactions.^1−4^ Formation of H_2_ in the interstellar medium (ISM) proceeds mostly on the surface of dust grains by recombination of two neutral hydrogen atoms or through channels like the interaction of cosmic rays with the ice coating of grains.^5^ Formation mechanisms and rates have been intensely studied.^1,2,6−8^ The state of interstellar clouds and their evolution are largely determined by the heating and cooling of H_2_.^9^ Despite its high abundance, molecular hydrogen is very hard to observe in the ISM due to its lack of a dipole moment; thus, neither microwave absorption nor emission occurs. Detection is possible via electronic excitations, which provide absorption bands in the far UV.^10^

Iron is the most abundant transition metal in the universe and may be present in the interstellar medium (ISM) in the form of dust grains or molecular complexes.^11−13^ However, only FeCN^12^ and FeO^14^ have been positively identified in the ISM. Laboratory spectra in search of further iron compounds have been provided for FeCO,^15^ FeCO^+^,^16^ Fe-PAHs,^17−19^ and Fe^+^(H_2_O),^20^ and high-level calculations are available for FeH^+^.^21^ We were able to observe the fundamental and overtone of the Fe–H stretching mode in Ar_2_FeH^+^,^22,23^ but argon tagging causes a substantial shift and broadening of the spectral band, which precludes comparison with observational data. Laboratory X-ray absorption spectra of the L_2,3_ edges of FeH^+^, in comparison with Cygnus X-1 observations, unfortunately did not provide any evidence for or against its presence in the ISM.^24^ Iron pseudocarbynes have been postulated by Tarakeshwar et al. to be present in the ISM since their infrared (IR) spectra are nearly identical to hydrocarbons in the absence of iron.^11^ Evidence for iron-containing nanoparticles has been found in the galactic ISM by Zhukovska et al.^13^ Bar-Num et al. found hints of iron hydride formation in matrix isolation studies and postulated that neutral iron hydride formation may proceed on ISM dust grains.^25^ Recent quantum chemical calculations by Fioroni and DeYonker indicated that Fe^+^ grafted onto the cosmic dust-grain siliceous surface can catalyze the reaction of two hydrogen atoms to form molecular hydrogen.^26^

Smith et al. deduced a bimolecular radiative association rate coefficient for the reaction of Na^+^ with H_2_ to form Na^+^(H_2_) of 4.4 × 10^–19^ cm^3^ s^–1^.^27^ Bieske and coworkers have performed infrared photodissociation spectroscopy on a series of M^+^(H_2_) and M^+^(D_2_) complexes.^28−33^ Complexation to the metal center turns the H–H/D–D stretching mode infrared-active so that the corresponding rovibrational band can be probed via H_2_/D_2_ loss. The Mg^+^(H_2_) complex was found to have vibrational properties of a T-shaped structure.^31^ Petrie and Dunbar estimated the radiative association rate of Mg^+^(H_2_) under interstellar medium conditions and concluded that this pathway is too slow to form appreciable amounts of Mg^+^(H_2_),^34^ mainly due to the very small Mg^+^–H_2_ binding energy of only 5.8 kJ/mol, according to their calculations. Recently, the vibrational spectroscopy of Cu^+^(H_2_)_4_ has been studied by the Asmis group in the region from 2500 to 7300 cm^–1^.^35^ Despite the low temperature of 10 K in the cryogenic ion trap, no rotational resolution could be obtained for the ≈60 cm^–1^ wide bands, suggesting a rather fluxional behavior of these complexes. Johnson and coworkers have used H_2_ and D_2_ as relatively weakly bound tags for cryogenic ion spectroscopy.^36,37^

The gas-phase ion chemistry of Fe^+^ with H_2_ was studied intensely by Elkind and Armentrout, who concluded that the low-lying excited ^4^F states of Fe^+^ are considerably more reactive than the ^6^D ground state.^38,39^ They also showed that the reaction of Fe^+^(^6^D) with H_2_ to form FeH^+^ faces a 0 K threshold of 2.34 ± 0.09 eV, ruling out its occurrence in the ISM. In view of the low pressure in the ISM, radiative association, (reaction 1), is the only plausible pathway.
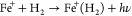
1

Bowers and coworkers established the binding energies of H_2_ ligands in Fe^+^(H_2_)*_n_* by high-pressure mass spectrometry equilibrium measurements.^40^ They found a significantly lower binding energy for the first ligand compared to the second—45 and 66 kJ/mol, respectively—which was explained by the energy needed for the promotion of the 4s electron to the 3d shell of Fe^+^. The electronic ground state of Fe^+^(H_2_)*_n_* complexes, therefore, has a quartet spin multiplicity. Formation of Fe^+^(H_2_) by ligand exchange of Fe^+^(CO) with H_2_ in the gas phase was reported by Mestdagh et al.^41^ Kobayashi et al. identified complexes Fe^+^(H_2_)_2,6_ in a solid hydrogen matrix by Mössbauer spectroscopy.^42^ However, no IR spectroscopic studies of Fe^+^(H_2_)*_n_* are available thus far.

Herein, we employ a high-resolution FT-ICR mass spectrometer combined with a tunable infrared laser in the range of 2240 to 4000 cm^–1^ to measure the infrared multiple photon dissociation (IRMPD) spectra of gas-phase Fe^+^(H_2_)_1,2_ and Fe^+^(D_2_)_1,2_ complexes. Quantum chemical calculations are performed to simulate the vibrational spectra and electronic transitions. The IRMPD spectrum was recorded via the loss of H_2_ and D_2_, respectively. We observed broad transitions with some narrower features assigned to contributions of the H–H and D–D stretches, respectively. The calculations indicate that the electronic transitions occur in the 3d shell of Fe^+^. Although coupling of electronic and vibrational degrees of freedom, together with the quantum nature of hydrogen motion, renders a quantitative simulation of the spectra extremely difficult, semiquantitative agreement between experiment and simulation is obtained, including the absolute absorption cross-sections.

## Experimental and Computational Methods

Infrared multiple photon dissociation (IRMPD) spectroscopy of Fe^+^(H_2_)_1,2_ and Fe^+^(D_2_)_1,2_ was performed on a modified 4.7 T Bruker Spectrospin CMS47X Fourier-transform ion cyclotron resonance (FT-ICR) mass spectrometer^43,44^ combined with an EKSPLA NT277 tunable optical parametric oscillator (OPO), as described previously.^45−47^ Cationic iron dihydrogen complexes were generated in a laser vaporization source. A Quantum Light Q2D33–1053 Nd:YLF laser (526.5 nm, max 25 mJ per pulse, 33.3 Hz) with H-SMART-SH-AT2 module for frequency doubling and attenuation (typically 5 mJ per pulse, 30 Hz) was focused on a rotating solid isotopically enriched iron disk (^56^Fe, STB Isotope Germany GmbH). The partly ionized metal vapor was entrained in a short gas pulse of helium seeded with hydrogen or deuterium, followed by supersonic expansion into a high vacuum. Guided by electrostatic ion optics, the complexes were trapped in the ICR cell, which was cooled to 80 K to minimize heating by ambient blackbody radiation.^48^ The ions of interest were mass selected in the ICR cell before irradiation with IR laser light. Infrared multiple photon dissociation (IRMPD) spectra were recorded over the full tuning range of the laser system, 2230 cm^–1^ to 4000 cm^–1^, where the characteristic H–H/D–D stretching mode is expected. The power of the tunable laser is presented in Figure S6. The tunable IR laser is operated at a repetition rate of 1000 Hz, with a bandwidth of less than 1 cm^–1^, as calibrated using a HighFinesse Laser Spectrum Analyzer IR-III. The beam diameter in the ICR cell at a distance of 5 m from the laser exit, after passing two focusing lenses, is estimated as 4 mm. The beam path is purged with dry nitrogen from a Domnick-Hunter nitrogen generator to minimize absorption by ambient air. One and two photon IRMPD cross-sections σ_1*hν*_ and σ_2*hν*_ were derived from the data, as described in detail before.^49^ The detection limit was estimated from the noise level of the mass spectra, which is the maximum potential fragment intensity that cannot be detected. The laser photon flux increases significantly from 2230 to 2800 cm^–1^, which is the reason for the elevated detection limit at low wavenumbers.

To complement the experimental results, geometric structures of the complexes and their IR spectra were calculated by using density functional theory (DFT) and coupled cluster methods, employing the B3LYP-D3 and CCSD methods together with the aug-cc-pVTZ basis set. Anharmonic frequency calculations were performed at the B3LYP-D3/aug-cc-pVTZ level of theory using second-order vibrational perturbation theory as implemented in Gaussian.^50^ Further, pGopher was employed to model the rotational contour of the rovibrational bands, using anharmonic vibrational frequencies and rotational constants from the calculations. All calculations in the electronic ground state were performed with the Gaussian 16 software package.^50,200^

To obtain a semiquantitative description of electronically excited states, we used complete active space self-consistent field (CASSCF) calculations followed by multireference configuration interaction (MRCI) and the inclusion of spin–orbit coupling using the Breit-Pauli operator, as implemented in Molpro.^51−53^ For Fe^+^(H_2_), we picked the active space of seven electrons in ten orbitals, further denoted as (7,10). The spectral shape is modeled with the reflection principle,^54−56^ using MRCI/aug-cc-pVDZ calculations with (7,10) and (7,7) active spaces for Fe^+^(H_2_) and Fe^+^(H_2_)_2_, respectively. A total of 2000 points taken at random from the quantum-harmonic Wigner distribution produced from B3LYP-D3/aug-cc-pVTZ calculations, neglecting vibrations with frequencies below 500 cm^–1^. Active space benchmarking is available in Figure S9. At each point, the calculated spectral line was broadened by a Gaussian function with a full width at half-maximum of 0.05 eV. We note that the reflection principle is a crude approximation in our case, as we ignore Franck–Condon overlaps that might produce a structured spectrum.

## Results and Discussion

### Calculations in the Electronic Ground State

**Figure 1:**
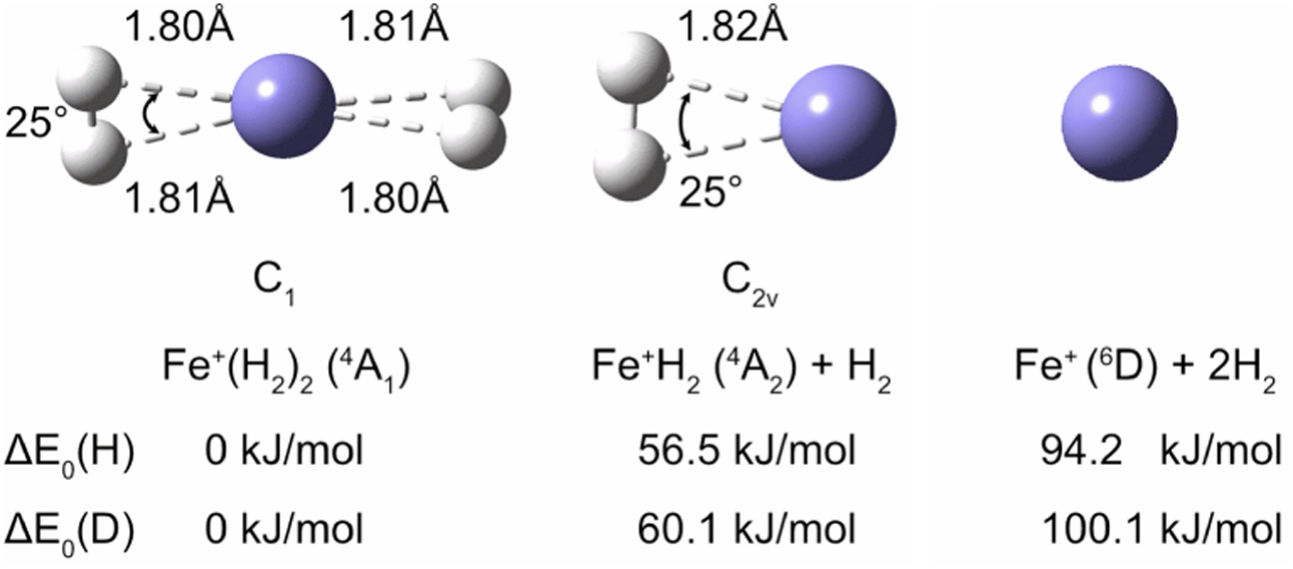
Lowest-energy structures of Fe^+^(H_2_) and Fe^+^(H_2_)_2_, optimized at the CCSD/aug-cc-pVTZ level of theory. Zero-point corrected relative energies, Δ*E*_0_(H) and Δ*E*_0_(D), are calculated from single-point energies at the CCSD(T)/aug-cc-pVTZ level, employing zero-point corrections at the CCSD/aug-cc-pVTZ level of theory.

To aid the interpretation of the experimental spectra, the geometry of Fe^+^(H_2_)_1,2_ was optimized at the CCSD/aug-cc-pVTZ level of theory. Figure 1 shows the equilibrium structures in C_2v_ symmetry for Fe^+^(H_2_). Due to the pseudo Jahn–Teller effect,^57^ the symmetry of Fe^+^(H_2_)_2_ is reduced to C_1_, staying still close to D_2d_. As pointed out before,^40^ the D_2h_ structure at 0.8 kJ/mol is only slightly higher in energy, but at the B3LYP-D3/aug-cc-pVTZ level, it has an imaginary frequency corresponding to the torsional motion of the two ligands. We note that the H–H and D–D frequencies are significantly blueshifted in the D_2h_ structure compared to those in the C_1_ structure. The calculated binding energies of the dihydrogen ligands, 37.7 and 56.5 kJ/mol for the first and second dihydrogen ligand, respectively, are slightly lower than the experimental values of 45 ± 3 kJ/mol and 66 ± 3 kJ/mol reported by Bowers and coworkers.^40^ They pointed out that the binding energy of the first H_2_ ligand is significantly smaller than that of the second, because the 4s electron of free Fe^+^ is promoted to the 3d subshell in the Fe^+^(H_2_) complex. Zero-point effects lead to slightly stronger binding in the deuterated species. Calculated harmonic and anharmonic frequencies are summarized in Table 1. The calculations show that the H–H stretching mode is moderately IR active in the Fe^+^(H_2_) complex. In Fe^+^(H_2_)_2_, the H–H stretching mode, with the two ligands oscillating in opposite phases, carries almost all the oscillator strength.

**Table 1:**
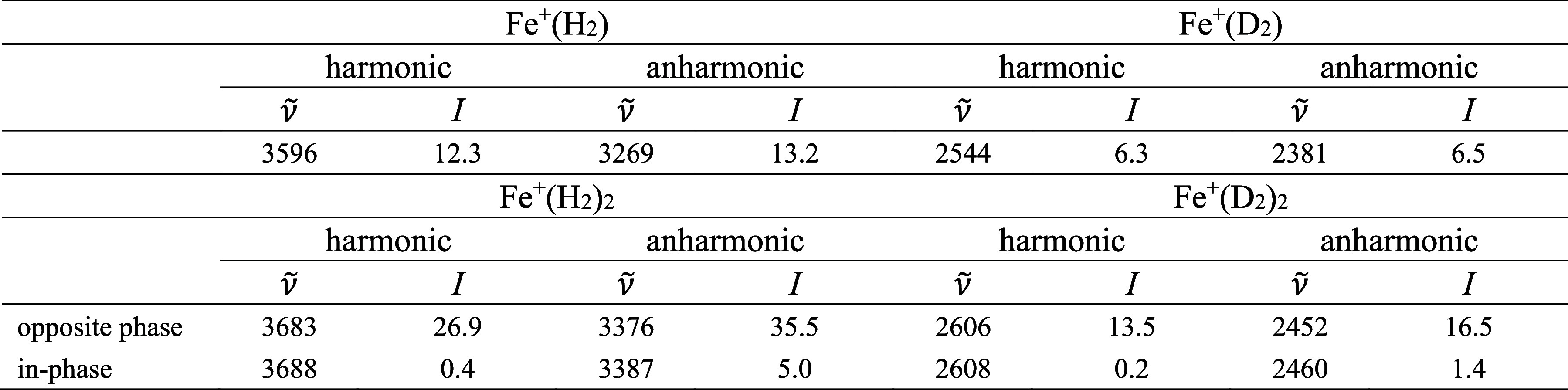
Harmonic and Anharmonic Frequencies  in cm^–1^ and Intensities *I* in km mol^–1^ of the H−H Stretching Modes in Fe^+^(H_2_)_1,2_ and Fe^+^(D_2_)_1,2_ as Determined at the B3LYP-D3/aug-cc-pVTZ Level of Theory without Applying a Scaling Factor^a^^,^^b^

a Results for all vibrational modes are provided in the Supporting Information.

b The benchmarking calculation are presented in Tables S3–S6.

### Experimental Spectra

**Figure 2:**
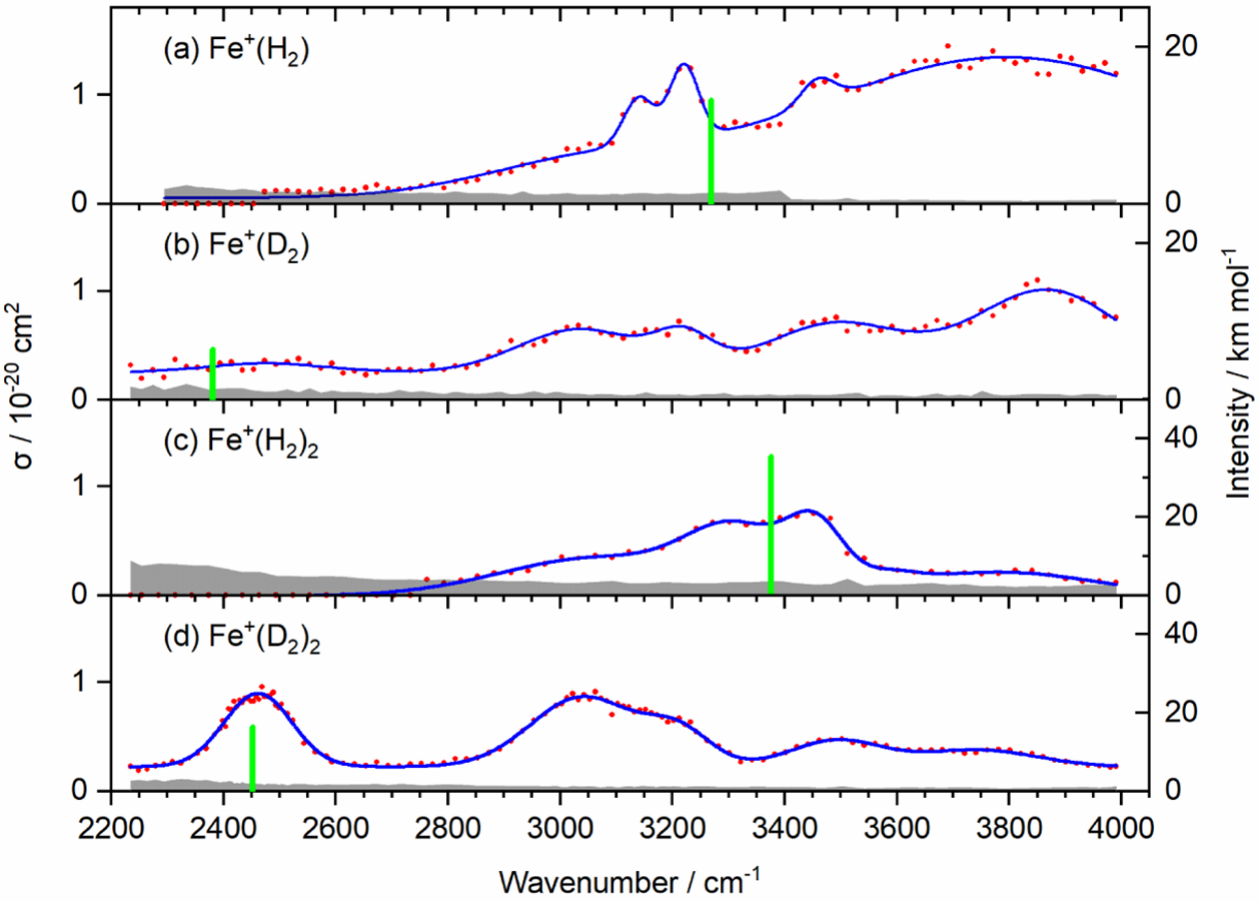
Experimental IRMPD spectra (red dots) of (a) Fe^+^(H_2_), (b) Fe^+^(D_2_), (c) Fe^+^(H_2_)_2_, and (d) Fe^+^(D_2_)_2_ at *T* ≈ 80 K. The blue curves are Gaussian cumulative fits. Position and intensity of the anharmonic H–H stretching frequency are shown as green bars, calculated at the B3LYP-D3/aug-cc-pVTZ level of theory. Gray shaded area denotes the detection limit.

IRMPD spectra of Fe^+^(H_2_)_1,2_ and Fe^+^(D_2_)_1,2_ are shown in Figure 2. To guide the eye, we fitted a minimum number of Gaussian peaks to the experimental data (see Table 2 and Figure S3). A 3-point running average is shown in Figure S4 for comparison. Broad, structureless bands, some of them several 100 cm^–1^ wide, dominate the spectra. With the known 3d^7^ configuration of Fe^+^ in these complexes,^40^ we assign most of these bands to rovibronic transitions, i.e., electronic excitations in the 3d^7^ subshell of Fe^+^ coupled to vibrational and rotational degrees of freedom. In the atomic ion, these transitions are forbidden, which is consistent with the small cross-section in the 10^–20^ cm^2^ range for the complexes.

**Table 2:** Position and Relative Intensity (10^–21^ cm^2^, in Parentheses) of Individual Gauss Peaks in the Cumulative Fit for Fe^+^(H_2_)*_n_* and Fe^+^(D_2_)*_n_* (*n* = 1,2) from Figure 2^a^^b^

	Gauss peak		Theory
	Position	fwhm	H–H stretch
Fe^+^(H_2_)	3041 (2.6)	441	
	3140 (4.3)	**64**	3269 (13.2)
	3220 (6.8)	**61**	
	3458 (2.2)	70	
	3794 (12.9)	870	
Fe^+^(D_2_)	2476 (0.8)	254	2381 (6.5)
	3035 (4.0)	259	
	3220 (2.9)	119	
	3492 (4.8)	286	
	3867 (7.6)	305	
Fe^+^(H_2_)_2_	3070 (3.5)	412	
	3314 (5.4)	**206**	3376 (35.5)
	3456 (5.5)	**119**	
	3588 (1.0)	115	
	3781 (2.2)	408	
Fe^+^(D_2_)_2_	2462 (6.7)	**142**	2452 (16.5)
	3041 (6.4)	219	
	3212 (3.0)	135	
	3493 (2.4)	193	
	3746 (1.5)	243	

a See the Supporting Information for benchmarking calculations

b Peaks assigned to the H–H stretching mode are printed in bold; closest matching calculated anharmonic frequency (cm^–1^) and intensity (km mol^–1^, in parentheses) for comparison, calculated using anharmonic frequency analysis on the B3LYP-D3/aug-cc-pVTZ level.

In addition to these electronic excitations, rovibrational transitions in the electronic ground state can be identified, as reported in boldface in Table 2. Comparison with the results of anharmonic calculations indicates the presence of a rovibrational band in Fe^+^(H_2_) (Figure 2a), which arises from the excitation of the H–H stretching mode in the electronic ground state. In the Fe^+^(D_2_) spectrum (Figure 2b), no clear feature is visible near the predicted position of the D–D stretching mode. For the complexes with two H_2_ ligands, two very broad, relatively weak maxima are present in the vicinity of the predicted band origin for Fe^+^(H_2_)_2_ (Figure 2c), which can again be assigned to the P and R branches of the rovibrational transition of the H–H stretching mode in the electronic ground state. A broad, strong band is observed for Fe^+^(D_2_)_2_ (Figure 2d), at the position predicted for the D–D stretching mode.

The absorption cross-sections in Figure 2 are calculated from fragment intensities, assuming a one-photon process, while two-photon cross-sections are available for comparison in Figure S1 and Table S2. The photon energy surpasses the calculated and experimental binding energies of H_2_ ligands in the complex only at the high-energy end of the spectra of the Fe^+^(H_2_) and Fe^+^(D_2_) complexes, while a second photon is required for dissociation at photon energies below the dissociation threshold. However, for the H_2_ complexes, we obtained photodissociation kinetics that exhibit first-order behavior (Figure S2), which indicates that the absorption of the first photon is the rate-limiting step. With the relatively low absorption cross-section in the 10^–20^ cm^2^ regime, the radiative emission lifetime is on the order of seconds, which affords dissociation after absorption of a second photon. The final dissociation event can either occur after excitation into the repulsive part of an electronically excited state above the dissociation threshold or by statistical unimolecular dissociation from any electronic state with a dissociation asymptote below the total energy of the system.

After absorption of the first photon with  > 2250 cm^–1^, the systems have acquired at least half the energy needed for dissociation. Due to the substantial anharmonicity of the vibrational modes and the availability of many low-lying electronic states, the photon energy is rapidly redistributed, regardless of whether the absorption occurred via a purely vibrational transition in the electronic ground state or a vibronic transition involving low-lying electronic states. The absorption cross-section for the second photon is therefore expected to be significantly higher than that for the first, which rationalizes why absorption of the first photon is rate limiting. The two-photon analysis shown for comparison in Figure S1 assumes identical cross-sections for two sequentially absorbed photons. We, therefore, conclude that the one-photon cross-sections shown in Figure 2 are more realistic than the two-photon values in Figure S1.

Based on these arguments, the absence of a clear feature for the D-D stretching mode of Fe^+^(D_2_) seems puzzling, since the spectrum of Fe^+^(D_2_)_2_ exhibits a pronounced band, and the bond dissociation energy of Fe^+^(D_2_) is only 40 kJ mol^–1^, compared to 60 kJ mol^–1^ for Fe^+^(D_2_)_2_. However, the ion signal for Fe^+^(D_2_) was significantly weaker than for Fe^+^(D_2_)_2_, and at the same time, the calculated absorption intensity is a factor of 2.5 smaller, which makes detection of the H–H stretching mode more challenging. In addition, we observe a broad, structureless transition in the relevant region, which is probably due to electronic excitation. Since the broad electronic bands also exhibit some intensity modulation, it may very well be that the D–D stretching mode coincides with a region of reduced intensity of the electronic transition, thus leveling out the expected weak feature.

### Excited-State Calculations and Electronic Structure

**Figure 3:**
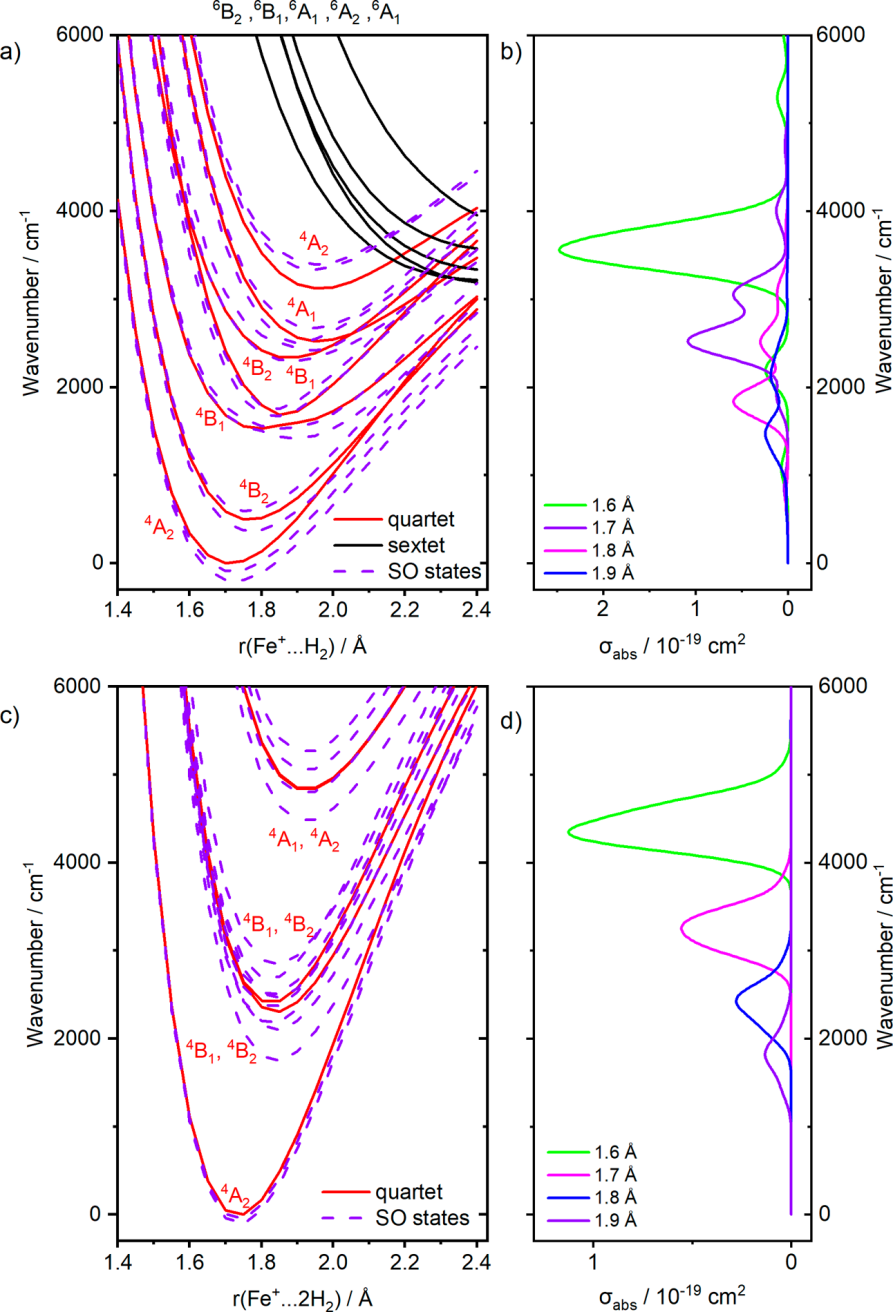
Potential energy curves in a) Fe^+^(H)_2_ and c) Fe^+^(H_2_)_2_ at the MRCI(7,10)/aug-cc-pVQZ level of theory. The quartet and sextet potentials are denoted by red and black solid curves, respectively. States including spin–orbit coupling are shown in dashed violet lines. C_2v_ point group was used for both Fe^+^(H)_2_ and Fe^+^(H_2_)_2_. b,d) Cross-section introduced by the reflection principle for a structure with a selected Fe^+^(H_2_)/Fe^+^(H_2_)_2_ distance, i.e., without averaging over the ground-state vibrational wave function, employing Gaussian broadening of 0.05 eV.

Figure 3a shows the electronic structure of the Fe^+^(H_2_) complex along the Fe^+^–H_2_ coordinate. In atomic Fe^+^, the term is ^6^D for the ground state and ^4^F for the first excited state without spin–orbit coupling. As mentioned above, H_2_ destabilizes the 4s orbitals of Fe^+^, preferring the lower-spin quartet electronic state (see Figure S7). At the same time, the presence of H_2_ lowers the symmetry to C_2v_, splitting the atomic term degeneracies. From the tentative ground state of the Fe^+^(H_2_) complex, ^4^A_2_, excitations into ^4^A_2_, ^4^B_1_, and ^4^B_2_ are symmetry allowed, with an excitation into the second ^4^B_1_ term being particularly intense.

The potential energy surfaces of the Fe^+^(H_2_) and Fe^+^(D_2_) complexes in Figure 3a show that the lowest energy dissociation asymptote arises from the sextet states, making dissociation near the energetic threshold spin-forbidden. However, the concept of two-state reactivity developed by Schwarz, Schröder, and Shaik implies that spin change in systems containing first-row transition metals, in particular iron, is not rate limiting.^58^ We can therefore assume that statistical unimolecular dissociation involving sextet states of Fe^+^ is indeed feasible. If dissociation occurs photochemically, i.e., by absorption of a second photon into a region of an excited-state potential energy surface that lies above the dissociation threshold, the curve crossings can afford predissociation, given the non-negligible spin–orbit coupling in iron-containing complexes. In either case, the observed fragment is Fe^+^. Since the mass spectrum does not reveal its quantum state, we have no experimental evidence for or against either one of these scenarios.

To illustrate the origin of the broad bands, we fixed the geometry of the Fe^+^(H_2_) complex at different Fe^+^–H_2_ distances and calculated the transition dipole moments for excitation from the lowest-lying spin–orbit component of the ^4^A_2_ ground state to all excited states, again including spin–orbit coupling. Each excited state then contributes a 0.05 eV wide Gaussian to the absorption spectrum at this fixed geometry. For example, the simulated spectrum at a fixed Fe^+^–H_2_ distance of 1.7 Å in Figure 3b reaches its maximum for the 2^4^B_1_ ← ^4^A_2_ transition, but excitations into other states add three more peaks. It should also be noted that the reflection principle does not account for discrete vibrational levels of electronic states.

The intensity of the spectrum depends strongly on the Fe^+^···H_2_ distance (Figure 3b). This can be understood in terms of the perturbation of the electronic structure of Fe^+^ by H_2_. For larger distances, Fe^+^ is almost unperturbed, and the selection rules of the atomic ion apply, i.e., the Laporte rule; see the weak spectrum for *r*(Fe^+^···H_2_) = 1.9 Å. When perturbed, transition dipole moment values, as well as splitting between states, increase, leading to absorption cross-sections on the order of ∼10^–19^ cm^2^, in overall agreement with the position of the experimental bands, as well as their intensity.

In the Fe^+^(H_2_)_2_ complex, a similar picture is obtained (Figure 3c). For computational efficiency, we fixed the symmetry to C_2v_ (a subgroup of D_2d_), and the distance between the two H_2_ ligands was changed simultaneously, which preserved symmetry. There is a pronounced minimum in the ^4^A_2_ state of the C_2v_ symmetry group, well separated from the ^4^B_1_/^4^B_2_ states, whose lowest spin–orbit component lies around 2000 cm^–1^. The transitions from ^4^A_2_ to ^4^B_1_/^4^B_2_ are symmetry allowed. Further ^4^A_1_/^4^A_2_ states lie well above 4000 cm^–1^ and thus do not contribute to the spectra in the experimental range. Again, at lower H_2_···Fe^+^···H_2_ distances (i.e., for a more disturbed system), peaks at higher energies as well as higher intensities are predicted. The 2^4^A_2_ state is responsible for pseudo-Jahn–Teller distortion and breaking of the D_2d_ symmetry.^57^ At an energy of almost 5000 cm^–1^ above the 1^4^A_2_ ground state, the pseudo-Jahn–Teller distortion caused by the 2^4^A_2_ state is small, which is in line with the small deviation from D_2d_ symmetry in the minimum geometry.

For direct comparison with the experiment, electronic spectra were modeled using Wigner sampling in a harmonic approximation, neglecting the vibrational resolution of the spectrum. To properly account for the effects of deuteration, which comprise narrower ground-state vibrational wave functions, spectra of the deuterated species were modeled separately. Since rovibrational transitions occur in parallel with the electronic absorption spectrum, we added rovibrational bands simulated with pGopher to obtain the complete spectrum, treating all complexes as symmetric tops;^59^ see Figure S5 and Table S7 for a detailed justification.

### Comparison of Experiment and Theory

**Figure 4:**
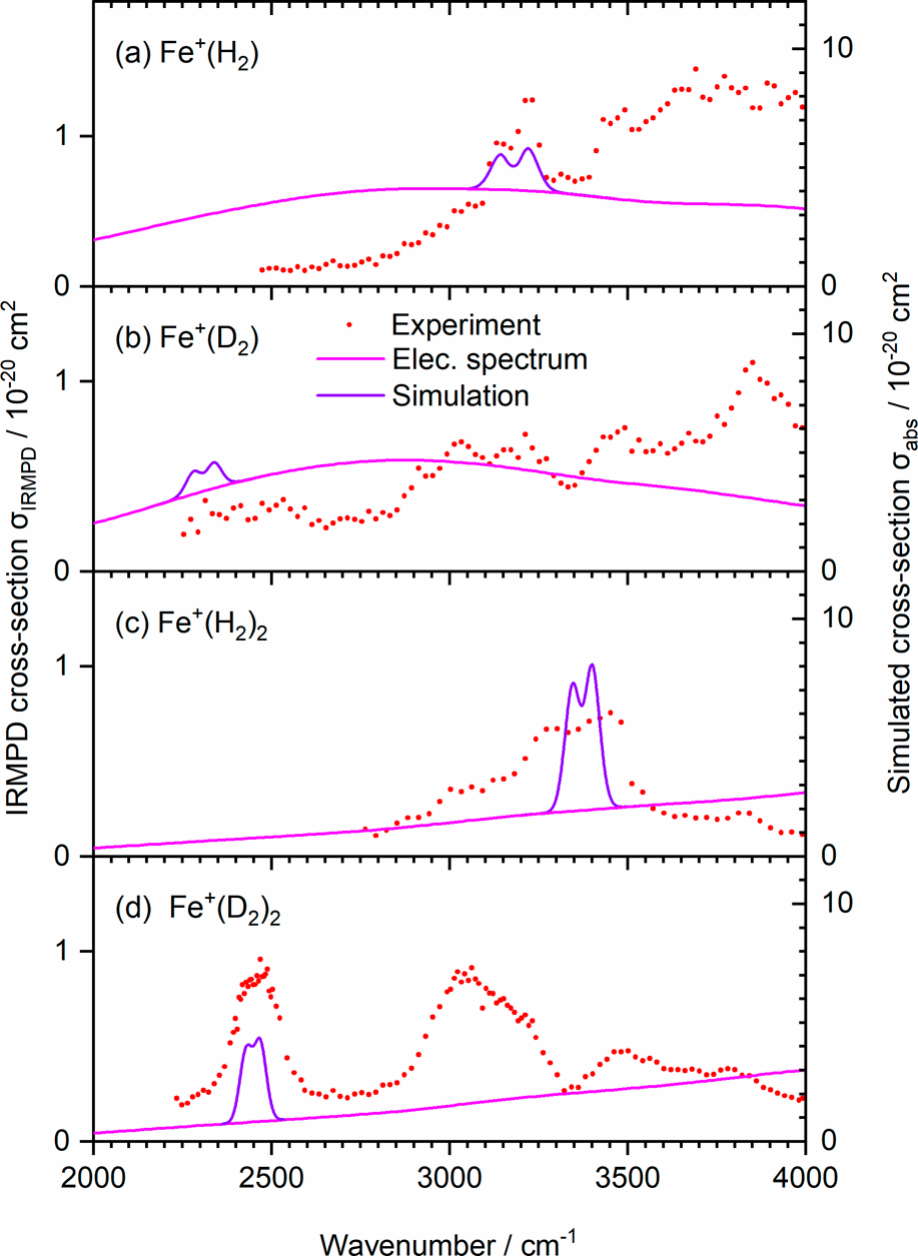
Combined simulated electronic spectrum of Fe^+^(H_2_)_1,2_ (pink line) and simulated rovibrational band (purple curve) of the H–H/D–D stretch in a) Fe^+^(H_2_), b) Fe^+^(D_2_), c) Fe^+^(H_2_)_2_, and d) Fe^+^(D_2_)_2_, in comparison with experimental IRMPD spectra (red dots).

Figure 4 shows the complete simulated spectra in comparison with our experimental data, and a wider range is shown in Figure S8. Absolute experimental cross-sections are systematically smaller than the calculated ones by roughly a factor of 10. The absolute experimental cross-sections are, first of all, subject to the uncertainty of the photon flux in the ICR cell, which we cannot measure directly. A second systematic problem is the multiple-photon nature of the dissociation. A possible explanation for the order-of-magnitude difference is a scenario where two photons are needed for dissociation, and IR emission occurs faster than the absorption of a second photon. For example, if only 10% of the complexes that absorb the first photon also absorb a second one before radiative cooling occurs, the experimental IRMPD cross-section is correspondingly smaller than the calculated absorption cross-section. Given the experimental uncertainties, we cannot quantify these effects, but the difference between measured σ_IRMPD_ and calculated σ_abs_ can be rationalized even with a perfect experiment. Due to these inherent problems, IRMPD yields^60^ are often reported instead of absolute cross-sections.

With respect to the spectral structure, our semiquantitative spectrum modeling approach predicts broad absorption bands induced by the perturbation of Fe^+^ electronic states by neighboring H_2_ molecules with large-amplitude zero-point motion relative to the metal center, with vibrational features of H_2_ stretching on top. Note that the width and position of the electronic spectrum are highly sensitive to the parameterization, such as the number of vibrational degrees of freedom considered in the Wigner sampling or the active space in MRCI.

The position of the rovibrational band in the Fe^+^(H_2_) spectrum (Figure 4a) is taken from the experimental spectrum as the average of the two Gaussians that are fitted to the putative P- and R-branches of the transition. The relative contributions of the electronic and rovibrational spectra are reasonably reproduced by the simulation. The envelope of the spectrum suggests a rotational temperature in the range of 300 K, the temperature of the ion source. The Fe^+^(D_2_) spectrum (Figure 4b) does not allow for the extraction of a rovibrational band origin. We therefore scaled the position in the Fe^+^(H_2_) spectrum by the D/H ratio obtained in the calculations. The simulated spectrum again shows a broad band, while the experimental spectrum seems to be more structured than the simulation. Also, there is no difference between the simulated electronic spectra of Fe^+^(H_2_) and Fe^+^(D_2_) while a considerable difference is observed in the experiment, suggesting that the main drawback of the modeling approach is the neglect of vibrational resolution.

The broad electronic band in the spectra of Fe^+^(H_2_)_2_ and Fe^+^(D_2_)_2_ (Figure 4c,d) reflects the increase in intensity at higher transition energies, as shown in Figure 3c,d. The simulated electronic spectra roughly match the experimental intensity but are, again, unable to reproduce the spectral shape due to the reflection principle approximation. The most prominent vibrational feature in the two spectra is the antisymmetric D–D stretch of the Fe^+^(D_2_)_2_ complex in Figure 4d. Here, the band position for the simulation is obtained from the Gaussian fit in Figures 2 and S3. The simulated R-branch is a bit more intense than the P-branch, and a similar feature seems to be present in the experiment. The experimental rovibrational band is significantly broader than the simulation, and we were not able to reproduce the experimental width by adjusting the simulation temperature. However, the significant blueshift of the D–D stretching mode in the D_2h_ transition structure mentioned above indicates that the frequency changes significantly along the torsional coordinate. We, therefore, think that the broadening is caused by the large-amplitude torsional motion of Fe^+^(D_2_)_2_. Since the zero-point amplitude is already substantial for this low-frequency mode, 44 cm^–1^ in the harmonic approximation (Table S1), this effect can explain the broadening of the band in pretty much the same way as the Ar–Fe–Ar bending mode broadens the Fe–H stretch in Ar_2_FeH^+^.^22^ In line with this argument, the experimental broadening is substantially more pronounced in Fe^+^(H_2_)_2_, to an extent where the P- and R-branch of the antisymmetric stretching mode can no longer be unequivocally assigned, as shown in Figure 4c. We, therefore, estimated the band origin for the simulation by scaling the corresponding Fe^+^(D_2_)_2_ frequency. The simulated rovibrational band is in the right position but much narrower than in the experiment. Since the amplitude of the zero-point motion in Fe^+^(H_2_)_2_ is significantly increased compared to Fe^+^(D_2_)_2_, the additional broadening is consistent with the blueshift of the H–H stretching mode along the torsional coordinate.

### Comparison with Other Metal–Dihydrogen Complexes

To understand the extent of activation of the H_2_ ligand in the iron-dihydrogen complexes studied here, it is instructive to compare our results with earlier works by the groups of Bowers, Bieske, and Asmis. H–H bond length, H–H stretch frequency, M^+^–H_2_ bond dissociation energy, and frequency shift are summarized in Table 3. As shown previously, the redshift of the H–H stretch correlates strongly with the bond dissociation energy. The Fe^+^–H_2_ bond dissociation energy is highest, especially if dissociation into the lowest-lying quartet state of Fe^+^, namely ^4^F_9/2_, is considered, which lies 1853 cm^–1^ above the ^6^D_9/2_ ground state. Consequently, the H–H bond length and the frequency shift are the largest of all systems investigated so far. The energy required for dissociation along the quartet asymptote also explains the slight blueshift of the H–H stretch in Fe^+^(H_2_)_2_ compared to Fe^+^(H_2_). The bonds are slightly weaker in the two-ligand system, leading to reduced activation of the H–H bond. The high binding energy and large frequency shift underline the relatively strong activation of the H_2_ ligands in Fe^+^(H_2_) and Fe^+^(H_2_)_2_, which arises from the significantly covalent character of the Fe^+^–H_2_ bond.

**Table 3:** Comparison of H–H Bond Lengths and H–H Stretch Frequencies of Bare H_2_ and D_2_ with Metal–Dihydrogen Complexes^a^

Complex	H–H bond length/Å	H–H frequency/cm^–1^	*D*_0_/cm^–1^	Δν_H–H_ /cm^–1^	Source
H_2_	0.741	4161	-	-	(62)
Li^+^(H_2_)	0.90^b^	4053	2275	108	(63), (64)
B^+^(H_2_)	0.756	3941	1329	221	(65)−^67^
Na^+^(H_2_)	0.746	4095	842/888^c^^,^^d^	66	(68)
Al^+^(H_2_)	0.746	4095	470	66	(69), (70)
Mg^+^(H_2_)	0.748	4055	416^d^	106	(31), (71)
Mn^+^(H_2_)	0.768	4049	660	112	(32), (72)
Fe^+^(H_2_)	0.779 (0.795)^e^	3180	3152^d^/5025^d^^,^^f^	981	This work
Fe^+^(H_2_)_2_	0.779 (0.790)^e^	3372	4723^d^	789	This work
Cu^+^(H_2_)_4_	0.766–0.774	3729	1760	432	(35), (73)
Zn^+^(H_2_)	0.774	3942^d^	1310	219	(28), (72)
Ag^+^(H_2_)	0.775	3756	3395^d^	406	(30)
D_2_	0.742	2994	-	-	(62)
Mg^+^(D_2_)	0.748	2918	-	76	(31)
Cr^+^(D_2_)	0.780	2779	-	215	(29), (74)
Fe^+^(D_2_)	0.779 (0.795)^e^	2309	3340^d^/5213^d^^,^^f^	685	This work
Fe^+^(D_2_)_2_	0.779 (0.790)^e^	2448	5024^d^	546	This work
Cu^+^(D_2_)_4_	0.766–0.774	2678	-	316	(35)
Zn^+^(D_2_)	0.758	2839	-	155	(28)

a Δν_H–H_ values denote the redshift relative to free H_2_ and D_2_ frequencies.

b Unrealistic value probably caused by large amplitude bending modes or hindered rotations.^63^

c Dissociation energies for Na^+^(H_2_) para and ortho.

d Theoretical values.

e The bond lengths are calculated at the CCSD/aug-cc-pVTZ level, with results at the B3LYP-D3/aug-cc-pVTZ level given in the parentheses.

f Dissociation to Fe^+^(^4^F_9/2_).

A closer look at the occupied valence molecular orbitals (MOs) (Figure S7) explains why the H_2_ ligand in Fe^+^(H_2_) experiences this high degree of activation. There are three MOs that contribute to the covalent character of the Fe^+^–H_2_ interaction. The lowest-lying MO 6a_1_ is dominated by the doubly occupied σ orbital of H_2_, which forms a three-center bonding MO with the d_*z*_^2^ orbital of Fe^+^. The antibonding counterpart is MO 7a_1_, which is dominated by the d_*z*_^2^ orbital of Fe^+^. The third MO contributing to the Fe^+^–H_2_ covalent interaction is the doubly occupied MO 3b_2_, which represents backdonation from the d_*xz*_ orbital of Fe^+^ into the σ* orbital of H_2_. This backdonation is responsible for the pronounced lengthening of the H–H bond and the strong redshift of the H_2_ vibrational mode.

To illustrate the consequences of this covalent character of the Fe^+^(H_2_) complex, we compare key geometric and spectroscopic parameters with those of Li^+^(H_2_). The interaction in the lithium complex is predominantly of the charge-induced dipole character, which, due to the weak polarizability of H_2_ perpendicular to the direction of the bond, results in a small bond dissociation energy and a long Li–H distance, *r*(Li–H). Also, the shift of the H_2_ stretching mode relative to that of free H_2_ is small. As a consequence, the antisymmetric stretching mode has a low frequency. The experimental fit of the Li^+^(H_2_) spectrum suggests that this mode may be better described as a hindered internal rotation of H_2_; i.e., Li^+^(H_2_) is a floppy system.^61^ If the bond in Fe^+^(H_2_) followed the same mechanism, the much larger ion radius of Fe^+^ would lead to an increased Fe–H distance, reduced binding energy, and reduced shift of the H–H stretching frequency. Clearly, the opposite is the case: the Fe–H distance is significantly reduced, the ligand binding energy *D*(Fe^+^–H_2_) is more than twice the value of the lithium complex, and the H–H stretching mode is strongly redshifted, as discussed in detail above. All this is caused by the pronounced covalent character of the interaction, with bonding contributions of σ and σ* orbitals of the hydrogen molecule interacting with iron d orbitals. The strong redshift of the H–H stretch is entirely due to the donation of electron density from the doubly occupied d_*xz*_ AO of Fe^+^ into the σ* orbital of H_2_. These covalent interactions make the molecule relatively stiff, as indicated by the unexpectedly high frequencies of the symmetric and antisymmetric Fe^+^–H_2_ stretching modes. Again, a naïve extrapolation from Li^+^(H_2_) would result in significantly smaller frequencies, since the reduced mass of the iron complex is larger and the force constants should be smaller in the complex with a larger ion radius. Clearly, the opposite is the case, the force constants of the two stretching modes increase 3–5 fold compared to Li^+^(H_2_). In turn, the stiffness of the Fe^+^(H_2_) complex justifies our simulation of the rovibrational spectra with rotational constants obtained from quantum chemistry Table 4.

**Table 4:** Comparison of Properties of H_2_, Li^+^(H_2_), and Fe^+^(H_2_) Calculated at the B3LYP-D3/aug-cc-pVTZ Level of Theory

Property	H_2_	Li^+^(H_2_)	Fe^+^(H_2_)
*D*_0_(M^+^–H_2_)/cm^–1^	-	2086	5881^a^
*r*(H–H)/Å	0.743	0.752	0.795
*r*(M–H)/Å	-	2.033	1.772
H–H stretch/cm^–1^	4413	4288	3596
M–H_2_ symmetric stretch/cm^–1^	-	339	812
M–H_2_ antisymmetric stretch/cm^–1^	-	682	1242

a Dissociation to Fe^+^(^4^F_9/2_): DFT result deviates from CCSD values in Table 3.

## Conclusions

IRMPD of gas-phase iron-dihydrogen complexes and their deuterium analogues reveals low-lying electronic transitions across the studied frequency range, which are due to excitations within the d subshell of Fe^+^. Parallel to the broad electronic spectra, rovibrational bands are observed that arise from the H–H stretching mode, which becomes weakly IR-active upon complex formation. High-level MRCI calculations, including spin–orbit splitting, qualitatively reproduce the electronic component of the experimental spectra. In particular, the small experimental cross-sections are replicated by theory. The shape of the H–H stretch rovibrational band in Fe^+^(H_2_) can be reproduced with a simulation at room temperature, while the corresponding features in the Fe^+^(H_2_)_2_ and Fe^+^(D_2_)_2_ spectra are substantially broadened. This broadening is attributed to the strong shift of the H–H and D–D antisymmetric stretching frequencies along the torsional mode, which has very low frequency and therefore a large zero-point amplitude. Fe^+^(H_2_) has the highest binding energy, largest H–H bond length, and largest frequency shift of the H–H stretching mode of all M^+^(H_2_) complexes studied so far. This underlines the covalent character of the interaction of the dihydrogen ligand with the iron center.

## Supporting Information Available

The two-photon analysis (Figure S1); the one-photon cross-sections (Figure S2); a minimum number of Gaussian peaks to the experimental data in Table 2 (Figure S3); a 3-point running average for comparison (Figure S4); simulation of the rovibrational bands of the H–H stretching mode of Fe^+^(H_2_)_1,2_ and Fe^+^(D_2_)_1,2_ with pGopher (Figure S5); the power of the tunable laser (Figure S6); the occupied valence molecular orbitals (MOs) explains why the H_2_ ligand in Fe^+^(H_2_) experiences this high degree of activation (Figure S7); theoretical absorption cross-section calculations for electronic transitions of Fe^+^(H_2_)_*n*_ and Fe^+^(D_2_)_*n*_, *n* = 1,2, in the spectral range; active space benchmarking (Figure S9); harmonic and anharmonic frequencies *ṽ* in cm^–1^ and intensities *I* in km mol^–1^ (Table S1); two-photon cross-sections for comparison (Table S2); H–H and D–D stretching frequencies in Fe^+^(H_2_), Fe^+^(D_2_), Fe^+^(H_2_)_2_, and Fe^+^(D_2_)_2_ harmonic and anharmonic theory (TableS S3–S6); Rotational constants calculated (Table S7); Cartesian coordinates and electronic energy of the optimized structure (PDF)
Original data shown in Figures 2–4, Figures S1–6, and Figure S8 (XLSX)
