# Supplementary material for: Infrared Multiple Photon Dissociation Spectroscopy of the H–H Stretching Mode and Low-Lying Electronic Transitions in Fe+(H2)1,2 and Fe+(D2)1,2
Source: J Phys Chem A. 2025 Apr 2;129(15):3455–65. doi: 10.1021/acs.jpca.5c00196 (PMC12010328; doi:10.1021/acs.jpca.5c00196)
Supplement: Supplementary file 1 — jp5c00196_si_001.pdf [file jp5c00196_si_001.pdf]

Supporting information

Infrared Multiple Photon Dissociation Spectroscopy of the H-H Stretching Mode and Low-Lying Electronic Transitions in $Fe^+(H_2)_{1,2}$ and $Fe^+(D_2)_{1,2}$

Shan Jin,[a] Marcos Juanes,[a,b] Christian van der Linde,[a] Milan Ončák[a,] and Martin K. Beyer[a,]**

[a]*Institut für Ionenphysik und Angewandte Physik, Universität Innsbruck, Technikerstraße 25, 6020 Innsbruck, Austria*

[b]*Departamento Química Física y Química Inorgánica, University of Valladolid, Paseo de Belén 7, 47011 Valladolid, Spain*

E-mail: milan.oncak@uibk.ac.at; martin.beyer@uibk.ac.at

ORCID:

Shan Jin	0000-0002-9460-853X
Marcos Juanes	0000-0002-7257-8632
Christian van der Linde	0000-0003-0493-820X
Milan Ončák	0000-0002-4801-3068
Martin K. Beyer	0000-0001-9373-9266

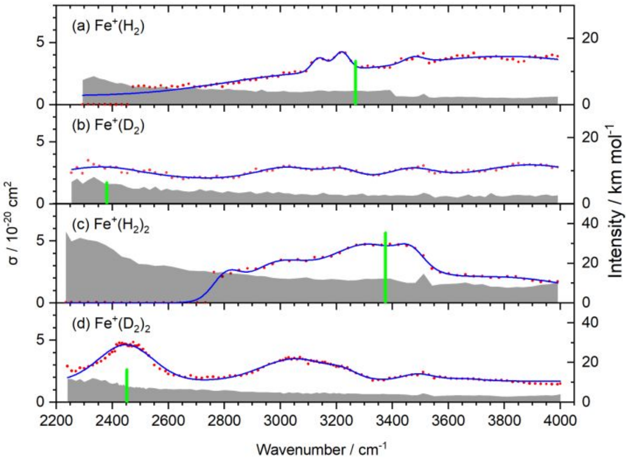

Figure S1. Experimental IRMPD spectra assuming a two-photon process (red dots) of (a) $Fe^+(H_2)$, (b) $Fe^+(D_2)$, (c) $Fe^+(H_2)_2$ and (d) $Fe^+(D_2)_2$ at T ≈ 80 K. The blue curves are Gaussian cumulative fits. Position and intensity of anharmonic H-H stretching frequency are shown as green bars, calculated at the B3LYP-D3/aug-cc-pVTZ level of theory. Grey shaded area denotes the detection limit.

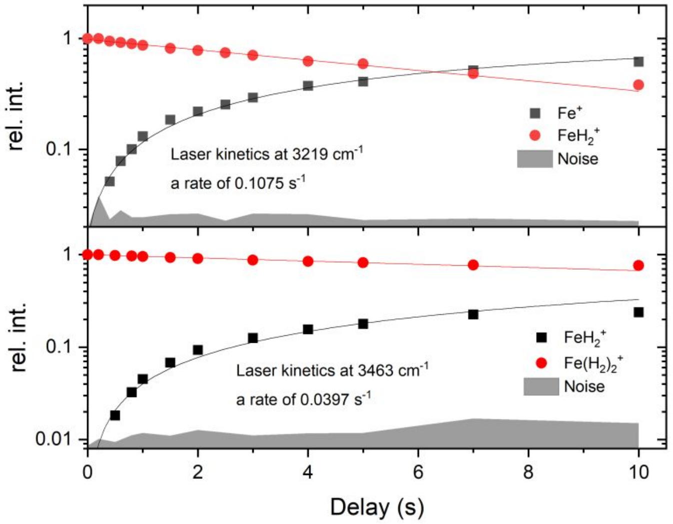

Figure S2. IRMPD kinetics of $Fe^+(H_2)$ and $Fe^+(H_2)_2$ at 3219 cm^{-1} and 3463 cm^{-1}, respectively, measured at 80 K.

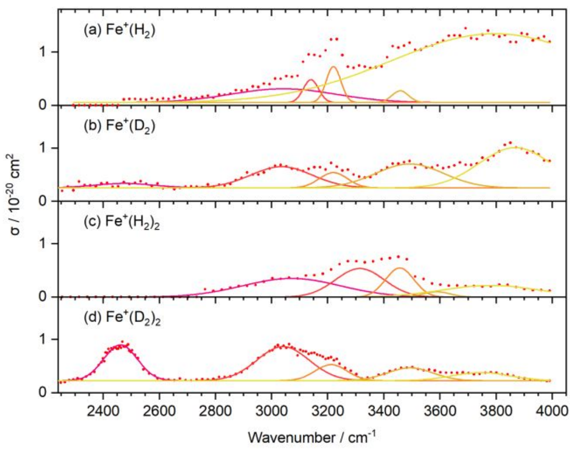

Figure S3. Contributions of individual Gaussians to the composite fit of the experimental spectra in Figure 2.

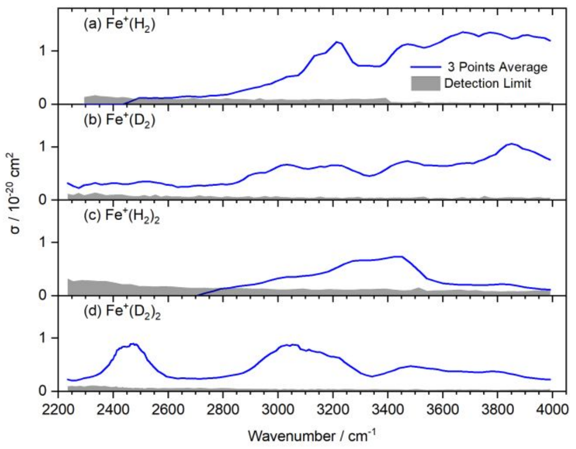

Figure S4. 3 points average of experiment spectra extracted from Figure 2. The average is running by Origin with the method of adjacent averaging.

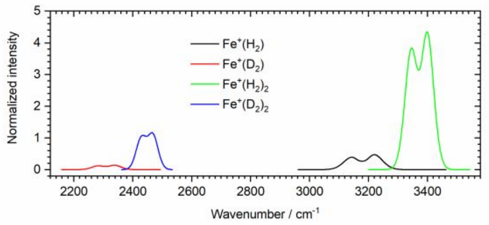

Figure S5. Simulation of the rovibrational bands of the H-H stretching mode of $Fe^+(H_2)_{1,2}$ and $Fe^+(D_2)_{1,2}$ with pGopher.[1] Rotational constants are listed in Table S7. The pGopher simulations were performed at 300 K with an arbitrary Gaussian broadening of 30 cm^{-1}.

Details on the pGopher simulation and justification of symmetric top approximation

According to Bunker and Jensen,[2] the parameter κ as defined in eq. (S1) can be used to decide which model to choose for the simulation of rotational band structure.

$$\kappa = (2B_e - A_e - C_e)/(A_e - C_e) \qquad \text{(S1)}$$

For a prolate top $\kappa = -1$, for an oblate top $\kappa = +1$, for an asymmetric top $-1 < \kappa < +1$. Rotational constants A_e, B_e, C_e are listed in Table S7, together with the parameter κ. In all cases, $\kappa \approx -1$, e.g. $\kappa(Fe^+(H_2)) = -0.994$, extremely close to a prolate top. We therefore choose the symmetric top model in pGopher for all four complexes. We use the geometric mean of the rotational constants B_e and C_e for the symmetric top. For $Fe^+(H_2)$ and $Fe^+(D_2)_2$ we shift the position of the band origin to match the experimental data as closely as possible, eg. Gaussian calculations predict H-H stretch in $Fe^+(H_2)$ and D-D stretch in $Fe^+(D_2)_2$ are at 3269 cm^{-1} and 2452 cm^{-1}, respectively. We shift the band origin in pGopher to 3180 cm^{-1} and 2448 cm^{-1} to match the experimental data as closely as possible. Such a match could not be obtained for $Fe^+(D_2)$ and $Fe^+(H_2)_2$, because no clear rovibrational band could be identified. In these cases, we instead infer the position of the band origin from the corresponding isotopologue, using a D/H wavenumber ratio of 0.726. Band origin in $Fe^+(D_2)$ and $Fe^+(H_2)_2$ pGopher simulations are 2309 cm^{-1} and 3372 cm^{-1}, respectively.

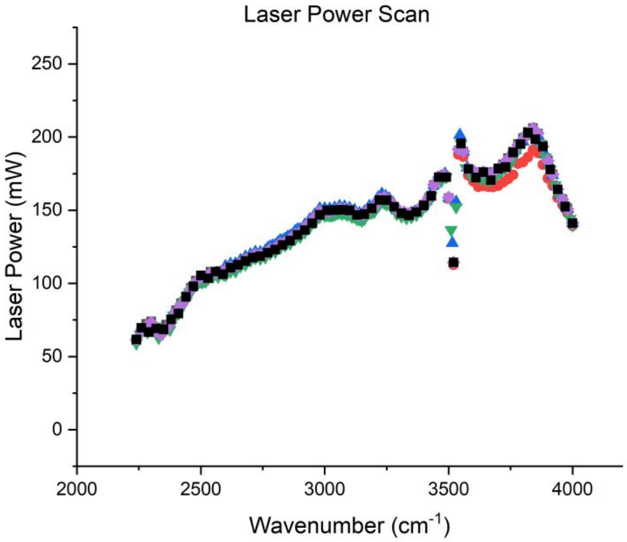

Figure S6. Laser power scans during the experimental running.

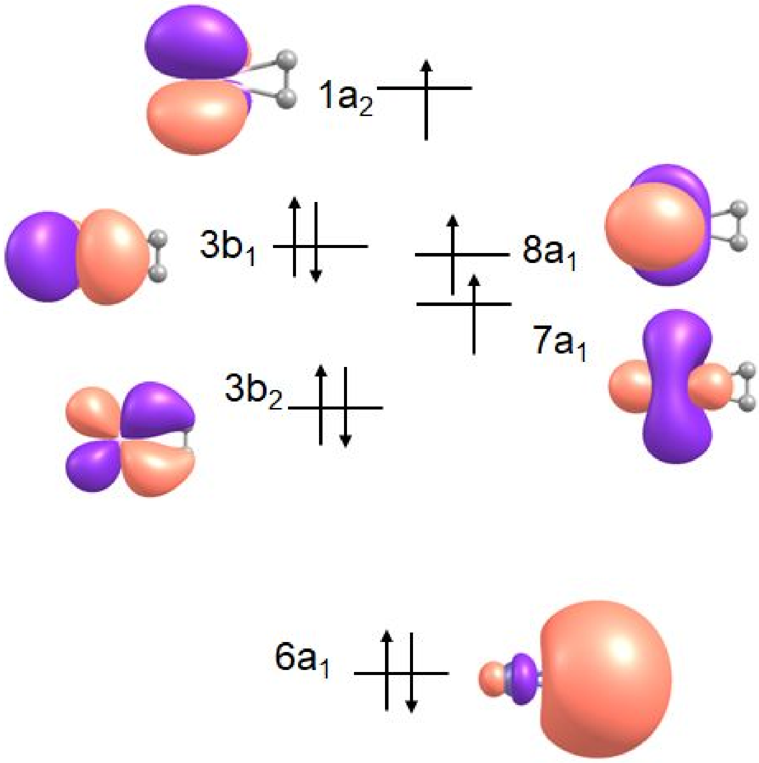

Figure S7. Electronic configuration of $Fe^{+}(H_2)$ in the $^{4}A_2$ ground state. Molecular orbitals were calculated at the CASSCF(7,10)/aug-cc-pVTZ level of theory averaging over 7 electronic states and are plotted with an isosurface value of 0.03.

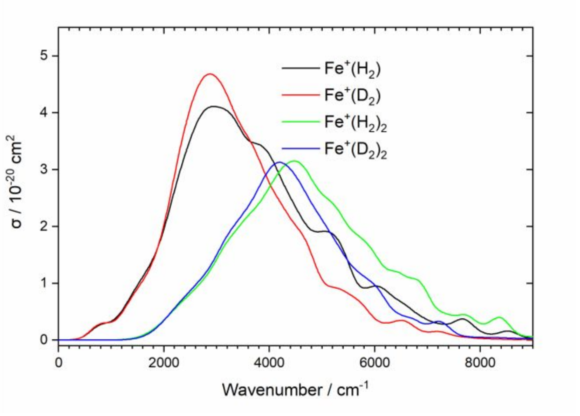

Figure S8. Theoretical absorption cross-section calculations for electronic transitions of $Fe^+(H_2)_n$ and $Fe^+(D_2)_n$, n = 1,2, in the spectral range of 0-9500 cm^{-1}. Calculations were performed at MRCI/aug-cc-pVDZ with (7,10) and (7,7) active spaces for $Fe^+(H_2)$ and $Fe^+(H_2)_2$, respectively.

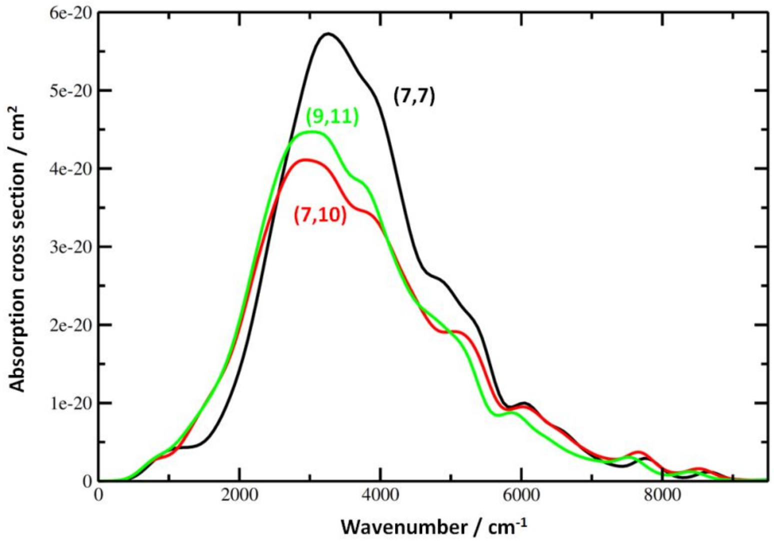

Figure S9. Comparison of theoretical absorption cross-section calculations for electronic transitions in $Fe^+(H_2)$. Calculations were performed at MRCI/aug-cc-pVDZ with (7,7), (7,10) and (9,11) active spaces.

Table S1. Harmonic and anharmonic frequencies $\tilde{\nu}$ in cm^{-1} and intensities I in km mol^{-1} in $Fe^+(H_2)_n$ and $Fe^+(D_2)_n$ (n = 1,2) as determined at the B3LYP-D3/aug-cc-pVTZ level of theory (without scaling). Unphysical frequencies predicted by anharmonic calculations are omitted from the table.

	$Fe^+(H_2)$				$Fe^+(D_2)$			
	harmonic		anharmonic		harmonic		anharmonic	
	$\tilde{\nu}$ / cm^{-1}	I / km mol^{-1}	$\tilde{\nu}$ / cm^{-1}	I / km mol^{-1}	$\tilde{\nu}$ / cm^{-1}	I / km mol^{-1}	$\tilde{\nu}$ / cm^{-1}	I / km mol^{-1}
Fe-H/Fe-D symmetric stretch	812	60.9	-	-	584	26.0	107	7.0
Fe-H/Fe-D asymmetric stretch	1242	10.5	961	7.2	879	4.7	739	3.6
H-H/D-D stretch	3596	12.3	3269	13.2	2544	6.3	2381	6.5
	$Fe^+(H_2)_2$				$Fe^+(D_2)_2$			
	harmonic		anharmonic		harmonic		Anharmonic	
	$\tilde{\nu}$ / cm^{-1}	I / km mol^{-1}	$\tilde{\nu}$ / cm^{-1}	I / km mol^{-1}	$\tilde{\nu}$ / cm^{-1}	I / km mol^{-1}	$\tilde{\nu}$ / cm^{-1}	I / km mol^{-1}
	60	0.8	-	-	44	0.0	-	-
	291	0.2	-	-	206	0.0	-	-
	359	6.9	386	108.6	262	1.6	301	13.8
Fe-H/Fe-D symmetric stretch	836	156.2	707	136.1	611	71.3	550	63.4
Fe-H/Fe-D symmetric stretch	875	0.0	765	0.1	619	0.0	570	0.1
Fe-H/Fe-D asymmetric stretch	1274	26.4	1134	6.0	902	12.2	840	3.3
Fe-H/Fe-D asymmetric stretch	1275	21.1	1115	79.5	903	9.9	836	25.6
H-H/D-D stretch opposite phase	3683	26.9	3376	35.5	2606	13.5	2452	16.5
H-H/D-D stretch in-phase	3688	0.4	3387	5.0	2608	0.2	2460	1.4

Table S2. Experimental band positions (2-photon process) for $Fe^+(H_2)_n$ and $Fe^+(D_2)_n$, n = 1,2, from Figure S1; vibrational frequencies (cm^{-1}) and intensities ($km\ mol^{-1}$, in parentheses) for $Fe^+(H_2)_n$ and $Fe^+(D_2)_n$ (n=1,2) in the H-H stretch region are given for comparison, calculated using anharmonic frequency analysis on the B3LYP-D3/aug-cc-pVTZ level.

	Experiment		Theory[a]
	position	fwhm	H-H stretch
$Fe^+(H_2)$	2980	615	
	3138	62	
	3219	64	3269 (13.2)
	3476	87	
	3807	1114	
$Fe^+(D_2)$	2359	347	2381 (6.5)
	3024	251	
	3220	136	
	3469	194	
	3893	406	
$Fe^+(H_2)_2$	2805	112	
	2979	262	
	3298	353	3376 (35.5)
	3466	109	
	3771	727	
$Fe^+(D_2)_2$	2448	228	2452 (16.5)
	3055	295	
	3227	112	
	3491	137	
	3655	163	

[a] See Supporting Information for benchmarking calculations.

Table S3. H-H stretching frequency in $Fe^{+}(H_2)$ harmonic and anharmonic theory as calculated using various methods and the aug-cc-pVTZ basis set (in cm^{-1}). The frequencies are unscaled.

Theory	B3LYP	B3LYP-D3	M06	BMK	CAM-B3LYP	ωB97XD	MP2	CCSD
Harmonic	3599	3596	3352	3824	3641	3626	4310	3850
Anharmonic	3288	3269	3175	3159	3359	3193	4561	-

Table S4. D-D stretching frequency in $Fe^{+}(D_2)$ harmonic and anharmonic theory as calculated using various methods and the aug-cc-pVTZ basis set (in cm^{-1}) the frequencies are unscaled.

Theory	B3LYP	B3LYP-D3	M06	BMK	CAM-B3LYP	ωB97XD	MP2	CCSD
Harmonic	2546	2544	2372	2705	2576	2565	3049	2721
Anharmonic	2391	2381	2283	2366	2434	2346	3392	-

Table S5. H-H stretching frequency in $Fe^{+}(H_2)_2$ harmonic and anharmonic theory as calculated using various methods and the aug-cc-pVTZ basis set (in cm^{-1}) the frequencies are unscaled.

Theory	Mode	B3LYP	B3LYP-D3	M06	CAM-B3LYP	ωB97XD	MP2	CCSD
Harmonic	opposite phase	3688	3683	3493	3716	3697	3832	3844
	in-phase	3693	3688	3496	3719	3701	3832	3848
Anharmonic	opposite phase	3425	3376	3374	3472	3535	-	-
	in-phase	3437	3387	3369	3483	3293	-	-

Table S6. D-D stretching frequency in $Fe^{+}(D_2)_2$ harmonic and anharmonic theory as calculated using various methods and the aug-cc-pVTZ basis set (in cm^{-1}) the frequencies are unscaled.

Theory	Mode	B3LYP	B3LYP-D3	M06	CAM-B3LYP	ωB97XD	MP2	CCSD
Harmonic	opposite phase	2609	2606	2474	2629	2616	2718	2719
	in-phase	2612	2608	2471	2631	2618	2723	2722
Anharmonic	opposite phase	2476	2452	2411	2505	2535	-	-
	in-phase	2483	2460	2413	2513	2405	-	-

Table S7. Rotational constants calculated at the B3LYP-D3/aug-cc-pVTZ level of theory.

	Rotational constants / MHz			Asymmetry Parameter
Ion	A_e	B_e	C_e	$\kappa = (2B_e-A_e-C_e)/(A_e-C_e)$
$Fe^{+}(H_2)$	1586830	87081	82550	-0.99398
$Fe^{+}(D_2)$	794025	45087	42664	-0.99355
$Fe^{+}(H_2)_2$	773851	40748	40731	-0.99995
$Fe^{+}(D_2)_2$	388195	20390	20384	-0.99997

Cartesian coordinates (Å) of optimized structures along with their electronic energy (Hartree)

Quartet spin multiplicity is used for $Fe^{+}(H_2)_{1,2}$ and $Fe^{+}(D_2)_{1,2}$.

$Fe^{+}(H_2)$, B3LYP/aug-cc-pVTZ

E = -1264.647602

Fe 0.000000 0.123282 0.000000

H -0.397535 -1.602662 -0.000000

H 0.397535 -1.602661 -0.000000

$Fe^{+}(H_2)$, B3LYP-D3/aug-cc-pVTZ

E = -1264.649031

Fe -0.000000 0.123367 -0.000000

H -0.397499 -1.603774 0.000000

H 0.397499 -1.603774 0.000000

$Fe^{+}(H_2)$, M06/aug-cc-pVTZ

E = -1264.536149

Fe 0.000000 0.000000 0.121061

H -0.000000 0.411345 -1.573790

H -0.000000 -0.411345 -1.573790

$Fe^{+}(H_2)$, BMK/aug-cc-pVTZ

E = -1264.055615

Fe 0.000000 0.126555 0.000000

H 0.391024 -1.645217 -0.000000

H -0.391024 -1.645214 0.000000

$Fe^{+}(H_2)$, Cam-B3LYP/aug-cc-pVTZ

E = -1264.678050

Fe 0.000000 0.122964 0.000000

H -0.397493 -1.598528 0.000000

H 0.397494 -1.598527 -0.000000

$Fe^{+}(H_2)$, ωB97XD/aug-cc-pVTZ

E = -1264.630983

Fe 0.000000 0.123256 0.000000

H -0.397605 -1.602329 0.000000

H 0.397605 -1.602328 -0.000000

$Fe^{+}(H_2)$, MP2/aug-cc-pVTZ

E = -1263.509123

Fe 0.000000 0.000000 0.162677

H 0.000000 0.375610 -2.114807

H -0.000000 -0.375610 -2.114807

$Fe^{+}(H_2)$, CCSD/aug-cc-pVTZ

E = -1263.547402

Fe -0.000000 0.000000 0.128692

H 0.000000 0.389558 -1.672998

H -0.000000 -0.389558 -1.672998

$Fe^{+}(D_2)$, B3LYP/aug-cc-pVTZ

E = -1264.647602

Fe 0.000000 0.123282 0.000000

H(iso=2) -0.397535 -1.602662 -0.000000

H(iso=2) 0.397535 -1.602661 -0.000000

$Fe^{+}(D_2)$, B3LYP-D3/aug-cc-pVTZ

E = -1264.649031

Fe 0.000000 0.123367 -0.000000

H(iso=2) -0.397499 -1.603774 0.000000

H(iso=2) 0.397499 -1.603774 0.000000

$Fe^{+}(D_2)$, M06/aug-cc-pVTZ

E = -1264.536149

Fe -0.000000 -0.000000 0.121061

H(iso=2) 0.000000 0.411345 -1.573790

H(iso=2) -0.000000 -0.411345 -1.573790

$Fe^{+}(D_2)$, BMK/aug-cc-pVTZ

E = -1264.055615

Fe -0.000000 0.126555 -0.000000

H(iso=2) -0.391023 -1.645216 0.000000

H(iso=2) 0.391024 -1.645215 0.000000

$Fe^{+}(D_2)$, Cam-B3LYP/aug-cc-pVTZ

E = -1264.678050

Fe -0.000000 0.122964 0.000000

H(iso=2) -0.397493 -1.598528 -0.000000

H(iso=2) 0.397494 -1.598527 -0.000000

$Fe^{+}(D_2)$, ωB97XD/aug-cc-pVTZ

E = -1264.630983

Fe -0.000000 0.123256 -0.000000

H(iso=2) -0.397605 -1.602329 -0.000000

H(iso=2) 0.397605 -1.602328 0.000000

$Fe^{+}(D_2)$, MP2/aug-cc-pVTZ

E = -1263.509123

Fe 0.000000 -0.000000 0.162677

H(iso=2) -0.000000 0.375610 -2.114807

H(iso=2) -0.000000 -0.375610 -2.114807

$Fe^{+}(D_2)$, CCSD/aug-cc-pVTZ

E = -1263.547402

Fe -0.000000 -0.000000 0.128692

H(iso=2) 0.000000 0.389558 -1.672998

H(iso=2) -0.000000 -0.389558 -1.672998

$Fe^{+}(H_2)_2$, B3LYP/aug-cc-pVTZ

E = -1265.857144

Fe -0.000000 -0.011063 -0.000115

H 1.713908 0.349542 -0.282270

H 1.750042 -0.205644 0.277867

H -1.713973 0.342574 0.290434

H -1.749976 -0.198840 -0.283034

$Fe^{+}(H_2)_2$, B3LYP-D3/aug-cc-pVTZ

E = -1265.860149

Fe 0.000000 -0.011082 0.000003

H 1.712780 0.346202 -0.286490

H 1.748948 -0.202135 0.280532

H -1.712778 0.346372 0.286293

H -1.748950 -0.202301 -0.280405

$Fe^+(H_2)_2$, M06/aug-cc-pVTZ

E = -1265.743680

Fe 0.000000 0.000000 0.000000

H -0.000000 0.404822 1.690076

H -0.000000 -0.404822 1.690076

H -0.404822 0.000000 -1.690076

H 0.404822 -0.000000 -1.690076

$Fe^+(H_2)_2$, Cam-B3LYP/aug-cc-pVTZ

E = -1265.880973

Fe 0.000000 -0.010747 -0.000006

H -1.707536 0.345078 -0.285862

H -1.742281 -0.205365 0.280238

H 1.742276 -0.204966 -0.280549

H 1.707541 0.344670 0.286334

$Fe^+(H_2)_2$, ωB97XD/aug-cc-pVTZ

E = -1265.836722

Fe 0.000000 0.010273 0.000000

H 1.728828 -0.071026 0.395359

H 1.728828 -0.071026 -0.395359

H -1.706400 -0.457218 -0.000000

H -1.751256 0.332183 -0.000000

$Fe^+(H_2)_2$, MP2/aug-cc-pVTZ

E = -1264.751248

Fe -0.000063 -0.011955 0.000000

H -1.761358 0.074812 0.389726

H -1.761358 0.074812 -0.389726

H 1.735758 0.469288 0.000000

H 1.788600 -0.308090 -0.000000

$Fe^+(H_2)_2$, CCSD/aug-cc-pVTZ

E = -1264.751251

Fe 0.000000 -0.000091 -0.012140

H 1.779860 0.282679 -0.186160

H 1.743768 -0.287378 0.343982

H -1.743647 0.290817 0.341520

H -1.779993 -0.283750 -0.183712

$Fe^+(D_2)_2$, B3LYP/aug-cc-pVTZ

E = -1265.857144

Fe 0.000000 -0.011064 -0.000003

H(iso=2) 1.713938 0.346184 -0.286255

H(iso=2) 1.750010 -0.202355 0.280397

H(iso=2) -1.713941 0.345979 0.286495

H(iso=2) -1.750008 -0.202155 -0.280549

$Fe^+(D_2)_2$, B3LYP-D3/aug-cc-pVTZ

E = -1265.860149

Fe 0.000000 0.011082 -0.000085

H(iso=2) 1.712805 -0.343662 0.289432

H(iso=2) 1.748921 0.199664 -0.282399

H(iso=2) -1.712752 -0.348888 -0.283321

H(iso=2) -1.748975 0.204765 0.278509

$Fe^+(D_2)_2$, M06/aug-cc-pVTZ

E = -1265.743680

Fe 0.000000 0.000000 0.000000

H(iso=2) 0.000000 0.404822 1.690076

H(iso=2) -0.000000 -0.404822 1.690076

H(iso=2) -0.404822 0.000000 -1.690076

H(iso=2) 0.404822 -0.000000 -1.690076

$Fe^{+}(D_2)_2$, Cam-B3LYP/aug-cc-pVTZ

E = -1265.880973

Fe 0.000000 -0.010747 0.000091

H(iso=2) 1.707575 0.341857 -0.289553

H(iso=2) 1.742242 -0.202223 0.282670

H(iso=2) -1.707502 0.347860 0.282610

H(iso=2) -1.742316 -0.208083 -0.278084

$Fe^{+}(D_2)_2$, ωB97XD/aug-cc-pVTZ

E = -1265.836722

Fe 0.000000 0.010273 0.000000

H(iso=2) 1.728828 -0.071026 0.395359

H(iso=2) 1.728828 -0.071026 -0.395359

H(iso=2) -1.706400 -0.457218 0.000000

H(iso=2) -1.751256 0.332183 0.000000

$Fe^{+}(D_2)_2$, MP2/aug-cc-pVTZ

E = -1264.751248

Fe -0.000063 -0.011955 0.000000

H(iso=2) -1.761358 0.074812 0.389726

H(iso=2) -1.761358 0.074812 -0.389726

H(iso=2) 1.735758 0.469289 -0.000000

H(iso=2) 1.788600 -0.308090 0.000000

$Fe^{+}(D_2)_2$, CCSD/aug-cc-pVTZ

E = -1264.751251

Fe 0.000001 -0.000260 -0.012137

H(iso=2) 1.779737 0.281666 -0.188431

H(iso=2) 1.743882 -0.284168 0.346235

H(iso=2) -1.743535 0.293986 0.339207

H(iso=2) -1.780117 -0.284722 -0.181439

References

(1) Western, C. M. PGOPHER: A program for simulating rotational, vibrational and electronic spectra. *J. Quant. Spectrosc. Radiat. Transf.* **2017**, *186*, 221–242. DOI: 10.1016/j.jqsrt.2016.04.010.

(2) Bunker, P. R.; Jensen, P. *Molecular symmetry and spectroscopy*, 2. ed.; NRC, Vol. 46853; NRC Research Press, 2006. DOI: 10.1139/9780660196282.
